# Adjustment of Measurements with Multiplicative Errors: Error Analysis, Estimates of the Variance of Unit Weight, and Effect on Volume Estimation from LiDAR-Type Digital Elevation Models

**DOI:** 10.3390/s140101249

**Published:** 2014-01-10

**Authors:** Yun Shi, Peiliang Xu, Junhuan Peng, Chuang Shi, Jingnan Liu

**Affiliations:** 1 School of Geomatics, Xi'an University of Science and Technology, Xi'an 710054, China; E-Mail: shiyun0908@hotmail.com; 2 Disaster Prevention Research Institute, Kyoto University, Uji, Kyoto 611-0011, Japan; 3 School of Land Science and Geomatics, China University of Geosciences, Beijing 100083, China; E-Mail: pengjunhuan@163.com; 4 GNSS Research Center, Wuhan University, Wuhan 430071, China; E-Mails: shi@whu.edu.cn (C.S.); jnliu@whu.edu.cn (J.L.)

**Keywords:** adjustment, multiplicative errors, error analysis, least squares, digital elevation model, landslide, hazard evaluation

## Abstract

Modern observation technology has verified that measurement errors can be proportional to the true values of measurements such as GPS, VLBI baselines and LiDAR. Observational models of this type are called multiplicative error models. This paper is to extend the work of Xu and Shimada published in 2000 on multiplicative error models to analytical error analysis of quantities of practical interest and estimates of the variance of unit weight. We analytically derive the variance-covariance matrices of the three least squares (LS) adjustments, the adjusted measurements and the corrections of measurements in multiplicative error models. For quality evaluation, we construct five estimators for the variance of unit weight in association of the three LS adjustment methods. Although LiDAR measurements are contaminated with multiplicative random errors, LiDAR-based digital elevation models (DEM) have been constructed as if they were of additive random errors. We will simulate a model landslide, which is assumed to be surveyed with LiDAR, and investigate the effect of LiDAR-type multiplicative error measurements on DEM construction and its effect on the estimate of landslide mass volume from the constructed DEM.

## 1. Introduction

Theory and methods of adjustment have been developed, both with the advance of measurement technology and with our deepened understanding of measurement errors. Although manufacturers of surveying instruments would always provide accuracy specifications [1], these specified numbers may not correctly reflect the nature of the errors of collected measurements. To understand the performance of a surveying instrument in practice, or equivalently, to obtain the accuracy of the instrument for a specific set of measurements, Airy [2] and Helmert [3] invented the theory and methods of variance component estimation, which has since become one of the important topics in statistics, geodesy and beyond (see e.g., [4–6]). If measurements are supposed to contain outliers, outlier detection and robust statistics are developed (see e.g., [7–13]). If prior information is available, Bayesian statistics and least squares collocation are accordingly borne naturally. To precisely determine the best trajectory of an object in motion, the theory and methods of optimal filtering, smoothing and control are incepted with the celebrated publication of Kalman [14]. Recently, with the advance of space observation technology such as global positioning system (GPS) and interferometric synthetic aperture radar (InSAR) (see e.g., [15–17]) and their wide applications, we have to handle observational models that contain real-valued and integer unknowns, which have been coined as mixed integer (linear) models by Xu [18].

Almost all theory and methods of adjustment have been developed on the basis of the following mathematical or functional model:

(1) y=f(β)+ε

where ***y*** is a vector of measurements. ***f***(***β***) is the linear or nonlinear functional, ***β*** is the vector of unknown parameters to be estimated. In the case of GPS and InSAR, the vector ***β*** may contain real-valued and integer unknowns, ***ε*** is the vector of random errors of the measurements ***y***. An important feature of the observational equation (1) is that the random errors ***ε*** will disturb the true values ***f***(***β***) of the measurements in the additive manner, implicitly indicating that the sizes of the random errors ***ε*** will not change with the sizes of measurements. In other words, ***ε*** has nothing to do with ***β*** or ***f***(***β***). However, in geodetic practice, we realize that some random errors of measurements change with the sizes of measurements themselves. For example, the accuracy of electronic distance measurements (EDM), GPS and/or VLBI baselines can be represented by using the following well-known formula:

(2) $$\sigma_{L}^{2}=a^{2}+b^{2}L^{2}$$

(see e.g., [1,19,20]), where $\sigma_{L}^{2}$ stands for the variance of the baseline of length *L*, *a* and *b* are positive constants. From the point of view of random error theory, the accuracy formula can be equivalently rewritten as follows:

(3) $$\varepsilon_{L}=\varepsilon_{a}+\varepsilon_{m}L$$

where *ε_L_* stands for the random error of the baseline *L*, *ε_a_* and *ε_m_* are the additive and multiplicative random errors, which are assumed to be statistically independent, with zero means and variances *a^2^* and *b*^2^, respectively. *ε_m_* in (3) is said to be a multiplicative error, since it will be propagated into the error of the baseline *L* through the baseline itself. As a result, this part of the error is proportional to the length of the baseline. The longer the baseline, the less accurate it is. However, the effect of the additive error *ε_a_* remains constant, irrelevant to the length of the baseline itself. As a second example, a SAR observational equation can be written as follows:

(4) $$y(i,j)=(1+\varepsilon_{ij})s(i,j)$$

(see e.g., [21–26]), where *s*(*I, j*) is the unknown signal to be estimated, *ε_ij_* is the random error with zero mean, and *y*(*i, j*) is the measured value of the signal *s*(*i, j*). The observational models (3) and (4) are obviously fundamentally different from (1). From this point of view, adjustment theory and methods, as developed on the basis of the model (1) (with only additive errors), cannot serve as a solid theoretical foundation to process measurements that are collected on the model (3) and/or (4).

The observational model (4) is called a multiplicative error model, which is widely known as a generalized linear model in statistics (see e.g., [27,28]). In engineering, it is also called a multiplicative speckle model (see e.g., [21,24]). The most widely used method in statistics to handle such a model is the quasi-likelihood method, which was invented by Wedderburn [27]. Quasi-likelihood has since become a standard method for parameter estimation in the model (4) and been widely applied in practice (see e.g., [29–32]). Mathematically, the method directly solves a set of nonlinear equations for the unknown parameters without any link to an objective function. As a result, Wedderburn [27] showed that the method is equivalent to maximum likelihood, if and only if the joint probability density function (pdf) of measurements is of exponential class. Unlike Wedderburn [27], Xu and Shimada [33] did not assume any pdf for the measurements with multiplicative errors. Instead, they directly started with the least squares (LS) method and derived the bias-corrected LS method for parameter estimation in the model (4).

A digital elevation model (DEM) is a numerical or digital representation of the topography of the Earth. A number of mathematical interpolators have been proposed for the construction of a DEM on the (implicit) assumption that the measurements are contaminated with additive random errors (see e.g., [34–36]). Recently, robust methods have also been proposed to construct a DEM by allowing the measurements to contain a certain percent of erroneous data (outliers) (see e.g., [37,38]). DEMs have found important applications in hazard assessment for disaster prevention and/or mitigation, for example, in landslide analysis [39,40], in construction of hazard maps [41] and to help reconstruct the history of hydrological and glacial events (see e.g., [42]). Practically, a DEM can be constructed by using remote sensing techniques such as (In)SAR and light detection and ranging (LiDAR). Although (In)SAR and LiDAR measurements have been well known to be of speckle (or multiplicative) noise, they have been used to construct a DEM as if these types of measurements were of additive random errors (see e.g., [37,39,43–45]). One of the purposes of this paper is to extend the methods of DEM construction to the case in which measurements are contaminated with multiplicative random errors and investigate their effect on the estimate of volume computed from LiDAR-type DEM, which can be important in practical hazard evaluation of landslides.

Although the errors of GPS, EDM and VLBI baselines and measurements of InSAR and LiDAR have been shown to be of multiplicative nature, almost nothing can be found in the geodetic and DEM literature, except for Xu *et al.* [46]. In this paper, we will limit ourselves to the model of multiplicative errors and substantially extend the work of Xu and Shimada [33]. We will not assume any pdf for the measurements with multiplicative errors. The paper is organized as follows: Section 2 will briefly outline three LS-based methods for parameter estimation. We will focus on analytical error analysis of all adjusted quantities of interest in Section 3, which further supplements the work of Xu and Shimada [33] in the sense that they mainly discussed parameter estimation and stochastic/numerical simulations. The error analysis of quantities other than model parameters is not covered by Xu *et al.* [46] either. In Section 4, we will derive the estimators of variance of unit weight in association with the three LS-based methods. Finally, we will simulate an example of DEM construction from LiDAR-type data contaminated with multiplicative random errors and investigate their effect on the estimate of volume from the constructed DEM.

## 2. Parameter Estimation in the Model with Multiplicative Errors

### 2.1. Representation of Models with Multiplicative Errors

As in the case of adjustment with additive random errors, given a set of measurements with multiplicative errors, we have the corresponding adjustment model as follows:

(5) $$y_{i}=f_{i}(\beta )(1+\varepsilon_{i}),(i=1,2,\ldots n)$$

(see e.g., [21,33]), or equivalently in vector form:

(6) y=f(β)⊙(1+ε)

where *y_i_* is a measurement, *f_i_*(*·*) is the functional model of the measurement *y_i_*, *ε_i_* is the random error with mean zero. If all these scalar quantities are represented in vector form, the corresponding vectors of the measurements *y_i_*, the functional models *f_i_*(*·*), and the random errors *ε_i_* can be denoted by ***y***, ***f***(***β***) and ***ε***, respectively. Here ***β*** is the t-dimensional vector of unknown parameters and ⊙ stands for the Hadamard product of matrices and/or vectors. In the observational equations (6), **1** stands for a vector with all its elements being equal to unity. Without loss of generality and for convenience of derivations, we will further assume that the elements of the random vector ***ε*** are all independent, namely, the variance-covariance matrix of ***ε*** is assumed to be equal to ***I****σ*^2^, where *I* is the identity matrix. If the functional models *f_i_*(*·*) are linear with respect to ***β*** namely, $f_{i}(\beta )=x_{i}^{T}\beta$, $x_{i}^{T}=(x_{i1},x_{i2}\ldots ,x_{it})$ is a *t*-dimensional vector, in this case, the model (6) with multiplicative errors can be rewritten as follows:

(7) y=(Xβ)⊙(1+ε)

where ***X*** = (*x*_1_,⋯, *x_n_*)*^T^*. It is obvious from the model (7) that the errors of the measurements ***y*** are proportional to the strengths of the signals. The stronger the signals (***Xβ***), the larger the errors of ***y***. Mathematically, the variance-covariance matrix **Σ***_y_* of the measurements ***y*** is the function of the unknown parameters ***β***. In the following, we will briefly outline three LS methods for parameter estimation, namely, the ordinary LS method, the weighted LS method and the bias-corrected weighted LS method.

### 2.2. The Three LS-Based Methods for Parameter Estimation

When the ordinary LS method is applied to the linear model (7) with multiplicative errors, we have the following minimization problem:

(8) $$\text{min}:\;{\text{F}}_{1}={\left(y-X\beta \right)}^{T}(y-X\beta )$$

It is well known that the optimal solution to (8) is the LS estimate of the parameters ***β***, *i.e.*

(9) $${\hat{\beta }}_{LS}={\left(X^{T}X\right)}^{-1}X^{T}y$$

It is trivial to prove that the ordinary LS estimate ***β̂_LS_*** is an unbiased estimate of ***β***.

If the random errors *ε*(*i = 1, 2, …, n*) in the model (7) are assumed to be statistically independent with the same variance, the measurements *y_i_*(*i = 1, 2, …, n*) are still statistically independent but are no longer of the same variance or weight. In this case, the weight matrix of the measurements *y_i_*(*i = 1, 2, …, n*) now depends on the unknown parameters ***β***. By denoting $D_{1}=\text{diag}(x_{i}^{T}\beta )$, with diag(·) standing for a diagonal matrix, we have the variance-covariance matrix **Σ***_y_* of the measurements ***y*** in (7) as follows:

(10) $$\sum_{y}=\text{E}(\varepsilon_{y}\varepsilon_{y}^{T})=D_{1}^{2}\sigma^{2}=D_{y}\sigma^{2}$$

where ***ε_y_*** = (***Xβ***)⊙***ε***. Since the elements of ***D***_1_ are the functions of the parameters ***β***, those of the variance-covariance matrix of the measurements ***y*** are also the functions of ***β***. If one replaces ***β*** in (10) with its approximate value, one can obtain an approximate variance-covariance matrix and then go on to estimate ***β*** with it. If an approximate value is sufficiently precise, the approximate estimate of ***β*** should be almost the same as the optimal estimate with a true variance-covariance matrix of the measurements. However, if approximate knowledge is not sufficient to obtain a precise approximation of **Σ*_y_***, the difference between the approximate and optimal estimates cannot be neglected, as can be seen in Xu *et al.* [46].

As a result of (10), we can apply the weighted LS method to (7) and obtain the following minimization problem:

(11) $$\text{min}:\;{\text{F}}_{2}={\left(y-X\beta \right)}^{T}D_{y}^{-1}(y-X\beta )$$

Differentiating (11) with respect to the unknown parameters ***β*** and letting it equal zero, we have:

(12) $$-2X^{T}{\hat{D}}_{y}^{-1}(y-X{\hat{\beta }}_{\mathrm{WLS}})+\left[\begin{array}{c} {\left(y-X{\hat{\beta }}_{\mathrm{WLS}}\right)}^{T}\frac{\partial {\hat{D}}_{y}^{-1}}{\partial \beta_{1}}(y-X{\hat{\beta }}_{\mathrm{WLS}})\\ \vdots \\ {\left(y-X{\hat{\beta }}_{\text{WLS}}\right)}^{T}\frac{\partial {\hat{D}}_{y}^{-1}}{\partial \beta_{t}}(y-X{\hat{\beta }}_{\text{WLS}}) \end{array}\right]=0$$

where ***β̂****_WLS_* stands for the weighted LS estimate of ***β***, and ***D̂****_y_* is the estimate of ***D****_y_*. According to the rule of matrix differentiation (see e.g., [47]), we have:

(13) $$\frac{\partial {\hat{D}}_{y}^{-1}}{\partial \beta_{i}}=-2\left[\begin{array}{ccc} \frac{{\text{x}}_{1i}}{{\left(x_{1}^{T}{\hat{\beta }}_{\text{WLS}}\right)}^{3}} & & \\ & \ddots & \\ & & \frac{x_{ni}}{{\left(x_{n}^{T}{\hat{\beta }}_{\text{WLS}}\right)}^{3}} \end{array}\right]=-2{\hat{P}}_{i}$$

Inserting (13) into (12) and after some arrangement, we can obtain the weighted LS estimate of ***β***, which is denoted by ***β̂****_WLS_* and given as follows:

(14) $${\hat{\beta }}_{\text{WLS}}={\left({\text{X}}^{T}{\hat{D}}_{\text{y}}^{-1}\text{X}\right)}^{-1}{\text{X}}^{T}{\hat{D}}_{y}^{-1}y+{\left(X^{T}{\hat{D}}_{y}^{-1}X\right)}^{-1}\left[\begin{array}{c} {\left(y-X{\hat{\beta }}_{\text{WLS}}\right)}^{T}{\hat{P}}_{1}(y-X{\hat{\beta }}_{\text{WLS}})\\ \vdots \\ {\left(y-X{\hat{\beta }}_{\text{WLS}}\right)}^{T}{\hat{P}}_{t}(y-X{\hat{\beta }}_{\text{WLS}}) \end{array}\right]$$

The weighted LS estimate ***β̂****_WLS_* of (14) is obviously nonlinear with respect to the measurements ***y***, even though the functional models $x_{i}^{T}\beta$ are linear in the unknown parameters ***β***, since the matrix ***D̂****_y_* and the elements of the second term on the right hand side of (14) are all the nonlinear functions of the weighted LS estimate ***β̂****_WLS_* itself. In general, we can only use numerical methods to solve for ***β̂****_WLS_*. Statistically, Xu and Shimada [33] proved that the weighted LS estimate ***β̂****_WLS_* is biased, even though $x_{i}^{T}\beta$ are linear; this is fundamentally different from the weighted LS estimation in a linear model with additive random errors. They also derived the bias of ***β̂****_WLS_*, and further proved that the bias of the weighted LS estimate ***β̂****_WLS_* is solely due to the fact that the variance-covariance matrix **Σ***_y_* of the measurements ***y*** is the function of the unknown parameters ***β***. In other words, the nonzero partial derivatives ***P̂****_i_* in (13) or (14) should be totally responsible for the bias of the weighted LS estimate ***β̂****_WLS_* [33,46]. In the case of linear models with additive random errors, since the variance-covariance matrix of measurements is independent of the unknown parameters, all ***P̂****_i_* are equal to zero. Therefore, they suggested removing the second term on the right hand of (14) and constructed an unbiased estimate as follows:

(15) $${\hat{\beta }}_{bc}={\left(X^{T}{\hat{D}}_{y}^{-1}X\right)}^{-1}X^{T}{\hat{D}}_{y}^{-1}y$$

where ***β̂****_bc_* is the bias-corrected weighted LS estimate.

We should note that formula (15) is the same as that derived by using the quasi-likelihood method, which nevertheless requires the class of exponential distributions in order for quasi-likelihood to be equivalent to maximum likelihood [27]. However, in this paper, we do not assume any distribution for the measurements ***y***. Actually, the estimate (15) is obtained solely from applying the weighted LS principle, indicating that more complicated adjustment with multiplicative errors can also be solved by using the conventional LS estimation principle.

## 3. Error Analysis of Quantities in the Model with Multiplicative Errors

Since Xu and Shimada [33] only focused on the estimation of parameters and numerical simulations to compute the biases and accuracy of the estimated parameters, we will complement their study by providing an analytical error analysis of the adjusted quantities. We should point out that error analysis is well documented in linear models with additive random errors, but nothing along the same line has ever been available in association with measurements contaminated by multiplicative errors, at least, to our best knowledge. Alternatively, one may carry out an error analysis with an approximate variance-covariance matrix of the measurements, as can be seen in geodetic adjustment of distance networks; but such an analysis cannot serve as a solid theoretical foundation of statistical inference in models with multiplicative errors, the extent of approximation will depend on the model itself, the stochastic model of measurements and prior knowledge on the unknown parameters. In this section, we will only assume the first and second central moments for the measurements ***y*** and will derive the accuracy formulae of the estimated quantities for the three LS-based estimation methods. We will limit ourselves to the first order approximation. We should note that some of the theoretical formulae to be given below may contain the unknown parameters, which should be substituted by their approximate values or estimates in practical computation.

### 3.1. Accuracy of the Estimated Parameters

Applying the error propagation law to the ordinary LS estimate (9) and after taking the variance-covariance matrix (10) into account, we can readily obtain the variance-covariance matrix of ***β̂****_LS_* as follows:

(16) $$\begin{array}{ll} \sum_{{\hat{\beta }}_{LS}} & ={\left(X^{T}X\right)}^{-1}X^{T}\sum_{y}X{\left(X^{T}X\right)}^{-1}\\ & ={\left(X^{T}X\right)}^{-1}X^{T}D_{1}^{2}X{\left(X^{T}X\right)}^{-1}\sigma^{2} \end{array}$$

Unlike the ordinary LS estimate ***β̂****_LS_*, (14) and (15) have clearly shown that both the weighted LS estimate ***β̂****_WLS_* and the bias-corrected weighted LS estimate ***β̂****_bc_* are nonlinear with respect to the measurements ***y***. In order to derive their accuracy formulae, we will have to represent ***β̂****_WLS_* and ***β̂****_bc_* in terms of the random errors ***ε*** and then follow the definition of variance to derive the variance-covariance matrices of ***β̂****_WLS_* and ***β̂****_bc_*. In this paper, we will limit ourselves to the first order approximation for accuracy assessment. Since the weighted LS estimate ***β̂****_WLS_* is not an unbiased estimate, we will also have to take its bias into account. In other words, we will have to compute the mean squared error matrix of ***β̂****_WLS_* for proper error analysis.

In order to derive the bias of the weighted LS estimate ***β̂****_WLS_*, Xu and Shimada [33] Taylor-expanded ***β̂****_WLS_* with respect to the random errors ***ε*** and then truncated the expansion up to the second order term, which is directly written as follows:

(17a) $${\hat{\beta }}_{\text{WLS}}=\beta +A\varepsilon +b_{\beta }$$

where:

(17b) $$A={\left(X^{T}{\hat{D}}_{y}^{-1}X\right)}^{-1}X^{T}D_{1}$$

and ***b****_β_* is a second order term of ***ε***, and its mathematical expectation is the bias of the weighted LS estimate.

We now apply the error propagation law to (17a) up to the first order approximation of ***ε*** and as a result, obtain the first order approximation of the variance-covariance matrix of the weighted LS estimate ***β̂****_WLS_* as follows:

(18) $$\sum_{{\hat{\beta }}_{\text{WLS}}}={\left(X^{T}D_{1}^{-1}X\right)}^{-1}\sigma^{2}$$

Denoting the bias of ***β̂****_WLS_* by E(***b****_β_*), we can finally obtain the mean squared error matrix as follows:

(19) $$\begin{array}{ll} M({\hat{\beta }}_{\text{WLS}}) & =\sum_{{\hat{\beta }}_{\text{WLS}}}+\text{E}(b_{\beta }){\left[\text{E}(b_{\beta })\right]}^{T}\\ & ={\left(X^{T}D_{y}^{-1}X\right)}^{-1}\sigma^{2}+{\left(X^{T}D_{y}^{-1}X\right)}^{-1}cc^{T}{\left(X^{T}D_{y}^{-1}X\right)}^{-1} \end{array}$$

where *c* **=** E(***b_β_***). Since the essential difference between the weighted LS estimate ***β̂****_WLS_* and the bias-corrected weighted LS estimate ***β̂****_bc_* is that ***β̂****_bc_* is unbiased up to the second order approximation. In other words, their first order representations in terms of ***ε*** are identical. Therefore, we can simply write the variance-covariance matrix of ***β̂****_bc_* below:

(20) $$\sum_{{\hat{\beta }}_{bc}}={\left(X^{T}D_{y}^{-1}X\right)}^{-1}\sigma^{2}$$

### 3.2. Accuracy of the Adjusted Measurements

By using the ordinary LS estimate ***β̂****_LS_* in (9), we can readily compute the corresponding LS adjusted values of the measurements:

(21a) $${\hat{y}}_{LS}=X{\left(X^{T}X\right)}^{-1}X^{T}y$$

According to the error propagation law, we can then obtain the variance-covariance matrix of the adjusted measurements as follows:

(21b) $$\sum_{{\hat{y}}_{LS}}=X{\left(X^{T}X\right)}^{-1}X^{T}D_{y}X{\left(X^{T}X\right)}^{-1}X^{T}\sigma^{2}$$

In the similar manner, based on the weighted LS estimate and the bias-corrected weighted LS estimate and by using the formulae (19) and (20), we can directly obtain the adjusted measurements computed from the weighted LS estimate and the bias-corrected weighted LS estimate, and their mean squared error (MSE) and variance-covariance matrices, respectively, as follows:

(22a) $${\hat{y}}_{\text{WLS}}=X{\hat{\beta }}_{\text{WLS}}$$

(22b) $$\begin{array}{ll} M({\hat{y}}_{\text{WLS}}) & =\text{X}\sum_{{\hat{\beta }}_{\text{WLS}}}{\text{X}}^{T}+XE(b_{\beta }){\left[X\text{E}(b_{\beta })\right]}^{T}\\ & =X{\left(X^{T}D_{y}^{-1}X\right)}^{-1}X^{T}\sigma^{2}+X{\left(X^{T}D_{y}^{-1}X\right)}^{-1}cc^{T}{\left(X^{T}D_{y}^{-1}X\right)}^{-1}X^{T} \end{array}$$

(23a) $${\hat{\text{y}}}_{bc}=X{\left(X^{T}{\hat{D}}_{y}^{-1}X\right)}^{-1}X^{T}{\hat{D}}_{y}^{-1}y$$

(23b) $$\sum_{{\hat{y}}_{bc}}=X{\left(X^{T}D_{y}^{-1}X\right)}^{-1}X^{T}\sigma^{2}$$

### 3.3. Accuracy of the Corrections of Measurements

As is well known, the corrections of measurements are important quantities in practical data processing and quality assessment/control. We denote the corrections of measurements as follows:

$$V=\hat{y}-y$$

where ***V*** is the correction vector of the measurements ***y***. By substituting (21a), (22a) and (23a) into the above formula, we can readily obtain the corrections of measurements with the ordinary LS, weighted LS and bias-corrected weighted LS estimates, respectively, as follows:

(24a) $$V_{LS}=[X{\left(X^{T}X\right)}^{-1}X^{T}-I]y$$

(25a) $$V_{\text{WLS}}=X{\hat{\beta }}_{\text{WLS}}-y$$

(26a) $$V_{bc}=[X{\left(X^{T}{\hat{D}}_{y}^{-1}X\right)}^{-1}X^{T}{\hat{D}}_{y}^{-1}-I]y$$

By linearizing (24a), (25a) and (26a) with respect to the random errors ***ε***, and after applying the error propagation law to the linearized corrections of measurements, we can then derive their corresponding variance-covariance matrices:

(24b) $$\sum_{V_{LS}}=[\text{X}{\left(X^{T}X\right)}^{-1}X^{T}-\text{I}]D_{y}[X{\left(X^{T}X\right)}^{-1}X^{T}-I]\sigma^{2}$$

(25b) $$\sum_{V_{\text{WLS}}}=[{\text{D}}_{y}-\text{X}{\left({\text{X}}^{T}D_{y}^{-1}X\right)}^{-1}X^{T}]\sigma^{2}$$

(26b) $$\sum_{V_{bc}}=[D_{y}-X{\left(X^{T}D_{y}^{-1}X\right)}^{-1}X^{T}]\sigma^{2}$$

Because the weighted LS estimate is biased, the corresponding corrections of measurements should also be biased. The bias of ***V****_WLS_* can be directly computed from the bias of the weighted LS estimate ***β̂****_WLS_*. Thus, for proper error evaluation, we have to compute the MSE matrix:

(27) $$\begin{array}{ll} M(V_{\text{WLS}}) & =\sum_{V_{\text{WLS}}}+XE(b_{\beta }){\left[XE(b_{\beta })\right]}^{T}\\ & =[{\text{D}}_{y}-\text{X}{\left(X^{T}{\text{D}}_{y}^{-1}X\right)}^{-1}X^{T}]\sigma^{2}+X{\left(X^{T}D_{y}^{-1}X\right)}^{-1}cc^{T}{\left(X^{T}D_{y}^{-1}X\right)}^{-1}X^{T} \end{array}$$

where ***M***(***V****_WLS_*)is the MSE matrix of ***V****_WLS_*.

### 3.4. The Covariances of the Adjusted Quantities

In practical data processing, we are also interested in computing the cross-covariance matrices of the original measurements, the estimated parameters, the adjusted measurements and the corrections of measurements. With the three LS-based methods in hand, if we follow the above lines of thought for the variance-covariance matrices of these quantities, we can then easily derive and obtain their covariances. If we further limit ourselves to the linear approximation in terms of the random errors ***ε***, then the weighted LS estimate and the bias-corrected weighted LS estimate should lead to the same covariance representations. Thus, by omitting the details of derivations, we simply list the cross-covariance matrices among the measurements, the estimated parameters, the adjusted measurements and the corrections of measurements for the ordinary LS and the bias-corrected weighted LS estimates in Table 1.

## 4. The Estimates of Variance of Unit Weight with the Three LS-Based Methods

The variance of unit weight is one of the most important quantities in statistical quality evaluation and hypothesis testing. Since the measurements are not equally weighted, the conventional estimator of the variance of unit weight by using the ordinary LS residuals of measurements and the redundant number of (*n–t*) is not unbiased [48]. To estimate the variance of unit weight, we have to start with the naïve or weighted sum of square of the corrections of measurements, find its mathematical expectation in terms of the variance of unit weight *σ*^2^ and then estimate *σ*^2^.In what follows, we will derive the estimates of the variance of unit weight in association with the three LS-based methods.

From the formula (24b), we can readily derive the mathematical expectation of the sum of square of the ordinary LS corrections of measurements:

(28a) $$\begin{array}{c} \text{E}(V_{LS}^{\text{T}}V_{LS})=tr\{[\text{X}{\left({\text{X}}^{\text{T}}\text{X}\right)}^{-1}{\text{X}}^{\text{T}}-\text{I}]D_{y}[X{\left(X^{T}X\right)}^{-1}X^{T}-\text{I}]\}\sigma^{2}\\ =tr\{{\text{D}}_{y}-X{\left(X^{T}X\right)}^{-1}X^{T}D_{y}X\}\sigma^{2} \end{array}$$

from which we can then construct the following estimate of the variance of unit weight:

(28b) $${\hat{\sigma }}_{LS1}^{2}=V_{LS}^{T}V_{LS}/r_{LS1}$$

where *r_LSl_* is the equivalent redundant number of measurements and given by

(28c) $$r_{LS1}=tr\{{\hat{D}}_{y}-{\left(X^{T}X\right)}^{-1}X^{T}{\hat{D}}_{y}X\}$$

If we use the weighted sum of square of the corrections of measurements in (28a), then the corresponding mathematical expectation can be rewritten as follows:

(29a) $$\begin{array}{ll} \text{E}({\text{V}}_{LS}^{T}{\hat{D}}_{y}^{-1}V_{LS}) & =tr\{\text{E}({\text{V}}_{LS}^{T}{\hat{D}}_{y}^{-1}V_{LS})\}\\ & =tr\{\text{E}({\hat{D}}_{y}^{-1}V_{LS}V_{LS}^{T})\}\\ & =[n-2t+tr\{D_{y}^{-1}-X{\left(X^{T}X\right)}^{-1}X^{T}D_{y}X{\left(X^{T}X\right)}^{-1}X^{T}\}]\sigma^{2} \end{array}$$

Accordingly, we can obtain the second estimate of the variance of unit weight from the ordinary LS estimate:

(29b) $${\hat{\sigma }}_{LS2}^{2}=V_{LS}^{T}{\hat{D}}_{y}^{-1}V_{LS}/r_{LS2}$$

where the second redundant number *r_LS_*_2_ of measurements is given by

(29c) $$r_{LS2}=n-2t+tr\{{\hat{D}}_{y}^{-1}\text{X}{\left({\text{X}}^{\text{T}}\text{X}\right)}^{-1}{\text{X}}^{\text{T}}{\hat{D}}_{y}\text{X}{\left(X^{\text{T}}X\right)}^{-1}X^{T}\}$$

For the weighted LS and bias-corrected weighted LS methods, if the bias of the weighted LS estimate is not significant, both methods should lead to almost the same estimates of the variance of unit weight. In the similar manner to the derivation of the estimate of the variance of unit weight in association with the ordinary LS method, we can readily use (25b) and (26b) to obtain the estimates of the variance of unit weight by using the weighted LS and the bias-corrected weighted LS estimates, which are simply listed, respectively, as follows:

(30) $${\hat{\sigma }}_{\text{WLS}1}^{2}=V_{\text{WLS}}^{T}{\hat{D}}_{y}^{-1}V_{\text{WLS}}/(n-t)$$

(31) $${\hat{\sigma }}_{bc}^{2}=V_{bc}^{T}{\hat{D}}_{y}^{-1}V_{bc}/(n-t)$$

where ${\hat{\sigma }}_{\text{WLS}1}^{2}$ and ${\hat{\sigma }}_{bc}^{2}$ stand for the estimates of the variance of unit weight by using the weighted LS and bias-corrected weighted LS estimates, respectively. If we remove the biases of the corrections of measurements due to the bias in the weighted LS estimate from the corrections of measurements, we can then obtain a new estimate of the variance of unit weight as follows:

(32) $${\hat{\sigma }}_{\text{WLS}2}^{2}={\left(V_{\text{WLS}}-\text{X}{\hat{b}}_{\beta }\right)}^{T}{\hat{D}}_{y}^{-1}(V_{\text{WLS}}-X{\hat{b}}_{\beta })/(n-t)$$

where ***b̂****_β_* is the bias of the weighted LS estimate.

## 5. Numerical Examples and Practical Effect on the Estimate of Volume from LiDAR-Type DEM

DEM has been playing an increasing role in hazard assessment (see e.g., [39–41]). A precise DEM will reduce the uncertainty of hazard mapping and could help make the operation and management of disaster rescue and support more focused. LiDAR has been widely used to construct DEM (see e.g., [37,39,40,43–45]). Although LiDAR measurements have been proved, both theoretically and experimentally, to be of multiplicative random errors (see e.g., [49–51]), they have been treated as if they were of additive error nature in DEM construction. Thus, this section is to serve two major purposes through numerical simulations: (i) to investigate the effect of LiDAR-type measurements on DEM construction; and (ii) to collectively use the error analysis and the estimates of the variance of unit weight given in the previous two sections to estimate the errors of the volume of landslide mass, since a precise estimate of the volume of a landslide from the constructed DEM can be important in practical hazard evaluation.

In this section, we will follow the website http://geology.com/usgs/landslides/with information on landslides and choose a rotational landslide model to simulate our numerical examples. A landslide of rotational type may create a curved surface. If the reader is interested in the worldwide damaging landslides in the 20th and 21st centuries, she/he may refer to the website http://landslides.usgs.gov/learning/majorls.php of the United States Geological Survey (USGS) for more information, with a landslide ranging from a few to many thousands millions cubic meter. The largest landslide in record seems to be the 1920 Haiyuan landslide, with an area of 50,000 square km and causing 100,000 plus deaths, due to the Haiyuan earthquake of magnitude M8.5, according to the above USGS website.

**Figure 1.**
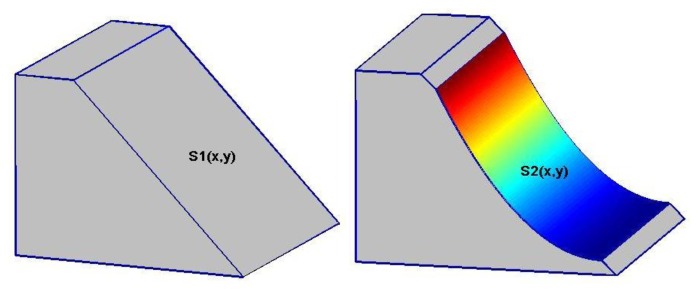
Illustrated model of landslide of rotational type. The left and right panels stand for the model mountains before and after the landslide, respectively.

To start with the simulation, we first assume a simple mountain of trapezoidal prism, with a slope of 50°, a height of 700 m and the length of 500 m. We then assume that a landslide occurs along the slope of the mountain, say due to a large earthquake. The design of landslide examples may be illustrated in Figure 1, with the left panel showing the original trapezoidal mountain and the right panel corresponding to the same mountain after the landslide. The volume of landslide mass can be computed by the following integration:

(33) $${\text{V}}_{sm}=\iint_{D}[S1(x,y)-S2(x,y)]\text{dxdy}$$

where *D* stand for the integration domain of the landslide surface *S2*(*x*, *y*) projected on the *xy* plane, the plane function *S1*(*x*, *y*) and the curved surface *S2*(*x*, *y*) are respectively represented as follows:

(34a) $$S1(x,y)=\beta_{10}+\beta_{11}x+\beta_{12}y$$

(34b) $$S2(x,y)=\beta_{20}+\beta_{21}x+\beta_{22}y+\beta_{23}x^{2}+\beta_{24}xy+\beta_{25}y^{2}$$

In this simulated example, the volume of the landslide mass is about 24.5686 million m^3^.

We now assume that DEMs are constructed by using airborne LiDAR before and after the landslide occurs, respectively. The height of flight is fixed at 900 m and the trajectory of the flight is assumed to be free of errors. For simplicity of simulation, we may also assume that the slope plane *S1*(*x*, *y*) before the landslide is sufficiently precise and treated as error-free. Since the surface function *S2*(*x*, *y*) has only six unknown parameters to be estimated for the complete reconstruction of the DEMs after the landslide, we use the uniform distribution to generate 30 LiDAR points for *S2*(*x*, *y*); this number of points is sufficiently large to estimate the variance of unit weight reliably. The standard deviation of multiplicative errors is equal to 0.5 per cent, *i.e.*, *σ* = 0.005, which is transformed into an equivalent standard deviation of unit weight of 1.268 m for the LiDAR ranging measurements.

With the simulated LiDAR data at hand, we can then estimate the surface *S2*(*x*, *y*) by using each of the three estimation methods given in Section 2 and further compute the estimated volume of the mass due to the landslide, which can be symbolically written as follows:

(35) $$\begin{array}{ll} {\hat{\text{V}}}_{\text{sm}}^{\text{j}} & ={\text{V}}_{0}+b_{20}{\hat{\beta }}_{20}^{j}+b_{21}{\hat{\beta }}_{21}^{j}+b_{22}{\hat{\beta }}_{22}^{j}+b_{23}{\hat{\beta }}_{23}^{j}+b_{24}{\hat{\beta }}_{24}^{j}+b_{25}{\hat{\beta }}_{25}^{j}\\ & ={\text{V}}_{0}+b_{2}^{T}{\hat{\beta }}_{2}^{j} \end{array}$$

where ${\hat{\text{V}}}_{\text{sm}}^{\text{j}}$ is the estimate of the volume V_sm_ of the landslide, the superscript j stands for each of the three estimation methods, namely, the ordinary LS, bias-corrected weighted LS and weighted LS methods. V_0_ and the coefficients *b_kl_* are constant, independent of the measurements and the estimated parameters but calculated by integrating the corresponding given functions of *S1*(*x*, *y*) and *S2*(*x*, *y*) without the unknown parameters ***β***_2_ in the domain *D*, and:

$$\begin{array}{l} b_{2}^{T}=(b_{20},b_{21},b_{22},b_{23},b_{24},b_{25})\\ {\hat{\beta }}_{2}^{j}={\left[{\hat{\beta }}_{20}^{j},{\hat{\beta }}_{21}^{j},{\hat{\beta }}_{22}^{j},{\hat{\beta }}_{23}^{j},{\hat{\beta }}_{24}^{j},{\hat{\beta }}_{25}^{j}\right]}^{T}. \end{array}$$

Listed in Table 2 are the true and estimated volumes of the simulated landslide mass by the three methods. It is clear from Table 2 that the bias-corrected weighted LS method leads to a difference of 38,664 m^3^ from the true value and performs better than the ordinary and weighted LS methods. The difference between the true volume and that estimated from the ordinary LS method is 80,790 m^3^, about the double of the difference by using the bias-corrected weighted LS method; a landslide with a size of this difference by itself is sufficiently large to cause severe damaging, when compared to the large landslides of the 20th and 21st centuries listed in the USGS website http://landslides.usgs.gov/learning/majorls.php. Nevertheless, the example has indicated that all the three methods can lead to good results, if the measurements are sufficiently precise.

In order to evaluate the errors of the estimated volume by each of the three methods, we apply the results of error analysis in Section 3 and the estimates of variance of unit weight in Section 4 to the estimate of volume (35) and readily obtain the error estimates for the estimated volumes of landslide mass. By the ordinary LS method one usually means to use (***A^T^ A***)^−1^ *σ̂^2^* to represent the accuracy, which is incorrect statistically. In fact, if one would do so, one would end up with a standard deviation of 65,130, which is only about 40 per cent of the correct standard deviation, as will be seen later. The correct error analysis for the ordinary LS method with multiplicative errors should follow Section 3, since too optimistic an incorrect estimate can be disastrous in disaster evaluation. For example, if the magnitude of the 2011 Tohoku mega-earthquake would have not been incorrectly under-estimated by more than one unit, most lives (even if not all) may have survived from the tsunami.

**Figure 2.**
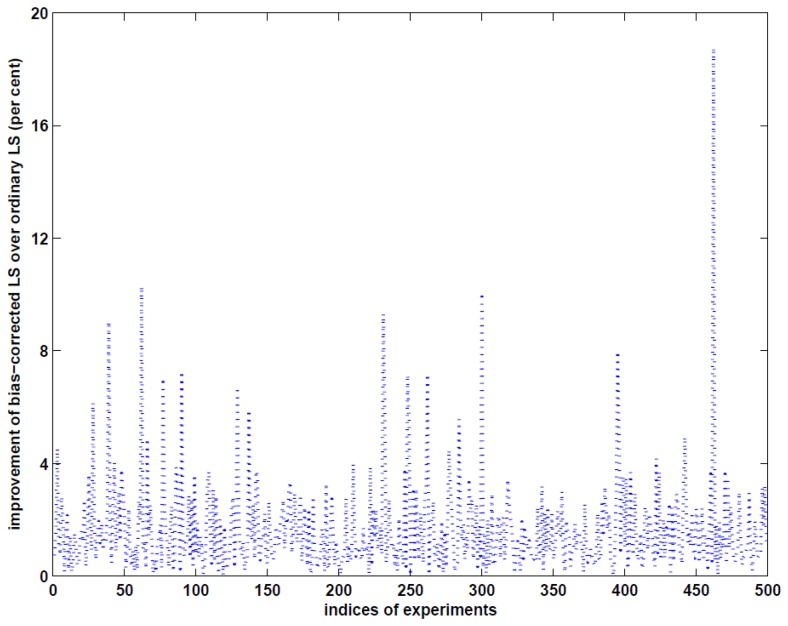
Accuracy improvement of the bias-corrected weighted LS method over the ordinary LS method (per cent) in the 500 independent random experiments.

More precisely, to estimate the accuracy of the estimated volume of the landslide, we first estimate the variance of unit weight by using (28b–c) for the ordinary LS method, (30) and (31) for the weighted LS and the bias-corrected weighted LS methods, respectively, and then compute the variance-covariance matrix of the estimated parameters in the case of the ordinary LS and bias-corrected weighted LS methods and the MSE matrix in the case of the weighted LS method. With these results at hand, we can finally apply the error propagation law to (35) and obtain the accuracy of the estimated volume of the landslide by each of the three methods. The MSE roots (RMSE) of the estimated volumes are given in row **RMSE** of Table 2. Obviously, the RMSE value by the bias-corrected weighted LS method is the smallest, which is nevertheless only better than the ordinary LS method by about 3.5 per cent in this specific example. The results of this example indicate that if LiDAR measurements are sufficiently precise, the three methods are able to precisely estimate the volume of landslide mass with sufficiently good accuracy. One may wonder whether the improvement of the bias-corrected weighted LS method over the ordinary LS method is marginally small. To answer this question, we simulate 500 independent examples with 30 different sampling points and repeat the above procedure to compute the RMSE values. The accuracy improvement of volume estimation with the bias-corrected weighted LS method over the ordinary LS method is shown in Figure 2. Depending on the locations of the measurements, the maximum improvement reaches about 19 per cent, which can have a significant consequence in hazard evaluation. Since the LiDAR measurements are assumed to be very precise, the biases of the estimated parameters from the weighted LS method are very small, as can be seen from the RMSE value in Table 2 in this example.

## 6. Concluding Remarks

Adjustment has been founded on the basis of observational models with additive random errors to process data in geoscience. The most important feature of an observational model with additive errors is that random errors do not change with the size of a signal or a measurement. However, with the advance of modern space observation technology, we realize that some of random errors change with the sizes of measurements, which may be attributed to the random nature of physical media along the path of observation. For example, GPS, VLBI and EDM baselines, InSAR and LiDAR measurements all show the feature of multiplicative random errors in the sense that their accuracy always contains one term that is proportional to the length of the baseline or the strength of signal. Theoretically speaking, the theory and methods developed on the basis of observational models with additive errors cannot meet the need of models with multiplicative errors. New theory and methods have to be developed accordingly. Xu and Shimada [33] demonstrated that the LS principle can be used to adjust observational models with multiplicative errors, unless the induced bias in the weighted LS estimate can be removed.

This paper has substantially extended the work by Xu and Shimada [33] by supplementing a complete error analysis for the quantities of interest. We have derived the cross-covariance matrices among such quantities. Since the variance of unit weight has been one of the most important quantities in statistical analysis of measurements and hypothesis testing, we have also constructed five estimators that correspond to the ordinary LS, the weighted LS and the bias-corrected weighted LS methods. Although LiDAR measurements have been known, theoretically and experimentally, to be of multiplicative error nature (see e.g., [37,39,43–45]), they have been used to reconstruct DEM as if they were of additive random errors. We have simulated an example of reconstructing DEM from LiDAR-type measurements and investigated the effect on volume estimation from the reconstructed DEM. The results have shown that all the three methods can well reconstruct the DEM, if measurements are sufficiently precise. The bias-corrected LS method can perform much better than the ordinary LS method, with a maximum improvement of about 19 per cent from 500 independent repetitions of the example experiment. Such an improvement could be important in hazard evaluation of landslides, for example.

## Figures and Tables

**Table 1.** Cross-covariance matrices among the measurements, the parameter estimates, the adjusted measurements and the corrections of measurements by the LS and bias-corrected weighted LS methods.

**Covariance**	**Ordinary LS Method**	**Bias-Corrected Weighted LS Method**
***cov***(***β̂,y***)	*(****X****^T^****X****)*^−1^***X****^T^****D****_y_σ*^2^	${\left(X^{T}D_{y}^{-1}X\right)}^{-1}X^{T}\sigma^{2}$
***cov***(***ŷ,y***)	***X***(***X****^T^****X***)^−1^***X****^T^****D****_y_σ*^2^	$X{\left(X^{T}D_{y}^{-1}X\right)}^{-1}X^{T}\sigma^{2}$
***cov***(***V,y***)	[***X****(****X****^T^****X****)*^−1^***X****^T^*−***I***]***D****_y_σ*^2^	$[X{\left(X^{T}D_{y}^{-1}X\right)}^{-1}X^{T}D_{y}^{-1}-I]D_{y}\sigma^{2}$
***cov***(***ŷ, β̂***)	***X****(****X****^T^****X****)*^−1^***X****^T^****D****_y_****X****(****X****^T^****X****)*^−1^*σ*^2^	$X{\left(X^{T}D_{y}^{-1}X\right)}^{-1}\sigma^{2}$
***cov***(***V, β̂***)	[***X****(****X****^T^****X****)*^−1^***X****^T^*−***I***]***D****_y_****X****(****X****^T^****X****)*^−1^*σ*^2^	**0**
***cov***(***V, ŷ***)	[***X****(****X****^T^****X****)*^−1^***X****^T^*−***I***]***D****_y_****X****(****X****^T^****X****)*^−1^***X****^T^σ*^2^	**0**

**Table 2.** The estimates of the volume of the simulated landslide mass. Also listed in this table are the roots of the MSE of the estimated volumes and the estimates of the standard deviation of unit weight.

**Methods**	**True**	**OLS**	**BCLS**	**WLS**
**Volume** (m^3^)	2.45686 × 10^7^	2.46494 × 10^7^	2.46073 × 10^7^	2.46105 × 10^7^
**RMSE** (m^3^)		1.640 × 10^5^	1.582 × 10^5^	1.583 × 10^5^
σ (m)	1.268	1.251	1.245	1.245

The abbreviations **OLS**, **BCLS** and **WLS** stand for the ordinary LS, bias-corrected and weighted LS methods, **Volume** for the volume of the landslide mass, and **RMSE** for the root mean squared errors of the estimated volumes of the landslide mass. σ is the standard deviation of unit weight.
